# Supplementary material for: Deciphering the late steps of rifamycin biosynthesis
Source: Nat Commun. 2018 Jun 14;9:2342. doi: 10.1038/s41467-018-04772-x (PMC6002545; doi:10.1038/s41467-018-04772-x)
Supplement: Supplementary file 1 — Supplementary Information [file 41467_2018_4772_MOESM1_ESM.pdf]

Supplementary Information

Deciphering the late steps of rifamycin biosynthesis

Qi *et al.*

TABLE OF CONTENTS

Supplementary Figures 4

Supplementary Figure 1 The putative rifamycin biosynthetic pathway 4

Supplementary Figure 2 Plasmid maps 5

Supplementary Figure 3 SDS-PAGE analysis of purified Rif16, Rif16$_{R84W}$, Rif15, Rif15a, and Rif15b 6

Supplementary Figure 4 The UV-visible absorption spectra of purified Rif16 7

Supplementary Figure 5 The inter-conversion of R-S and R-SV 8

Supplementary Figure 6 The spontaneous oxidation of R-SV to R-S in the presence of different divalent metal ions 9

Supplementary Figure 7 Protein sequence alignment of Rif15 and six other transketolases 11

Supplementary Figure 8 The high resolution mass spectrum of R-L 12

Supplementary Figure 9 ^{1}H NMR spectra of R-S, R-B, and R-L 13

Supplementary Figure 10 ^{13}C NMR and DEPT135 spectra of R-L 14

Supplementary Figure 11 HSQC spectrum of R-L 15

Supplementary Figure 12 ^{1}H-^{1}H COSY spectrum of R-L 16

Supplementary Figure 13 HMBC spectrum of R-L 17

Supplementary Figure 14 The activity of the transketolase Rif15 with different C_2 donors 18

Supplementary Figure 15 The high resolution mass spectrum of biosynthesized R-B 19

Supplementary Figure 16 Multiple protein sequence alignment between Rif16 and other P450 enzymes with their substrates different in size and shape 20

Supplementary Figure 17 Structures of Rif16 21

Supplementary Figure 18 HPLC-HRMS analysis of the transient intermediate (R-O) between R-L and R-B 22

Supplementary Figure 19 The high resolution mass spectra of the ^{13}C labeled R-L and R-B 23

Supplementary Figure 20 The substrate binding curve R-L toward Rif16 24

Supplementary Figure 21 UV-visible absorption spectra of purified Rif16$_{R84W}$ 25

Supplementary Figure 22 HPLC analysis of the reactions catalyzed by Rif16$_{R84W}$ 26

Supplementary Figure 23 HPLC analysis of R-L degradation ... 27
Supplementary Figure 24 Protein sequence alignment of Rif16 and nine other P450s ... 28
Supplementary Tables ... 30
Supplementary Table 1 The nucleotide sequences of *rif15* and *rif16* ... 30
Supplementary Table 2 ^{1}H (600 MHz) and ^{13}C (150 MHz) NMR data for R-L and R-B in CD_3OD ... 31
Supplementary Table 3 Amino acid sequence similarity and identity of Rif15a, Rif15b and Rif16 with other similar proteins ... 32
Supplementary Table 4 Oligonucleotide primers used in this study ... 33
Supplementary Table 5 Data collection and refinement statistics for Rif16 structures ... 34
Supplementary References ... 35

Supplementary Figures

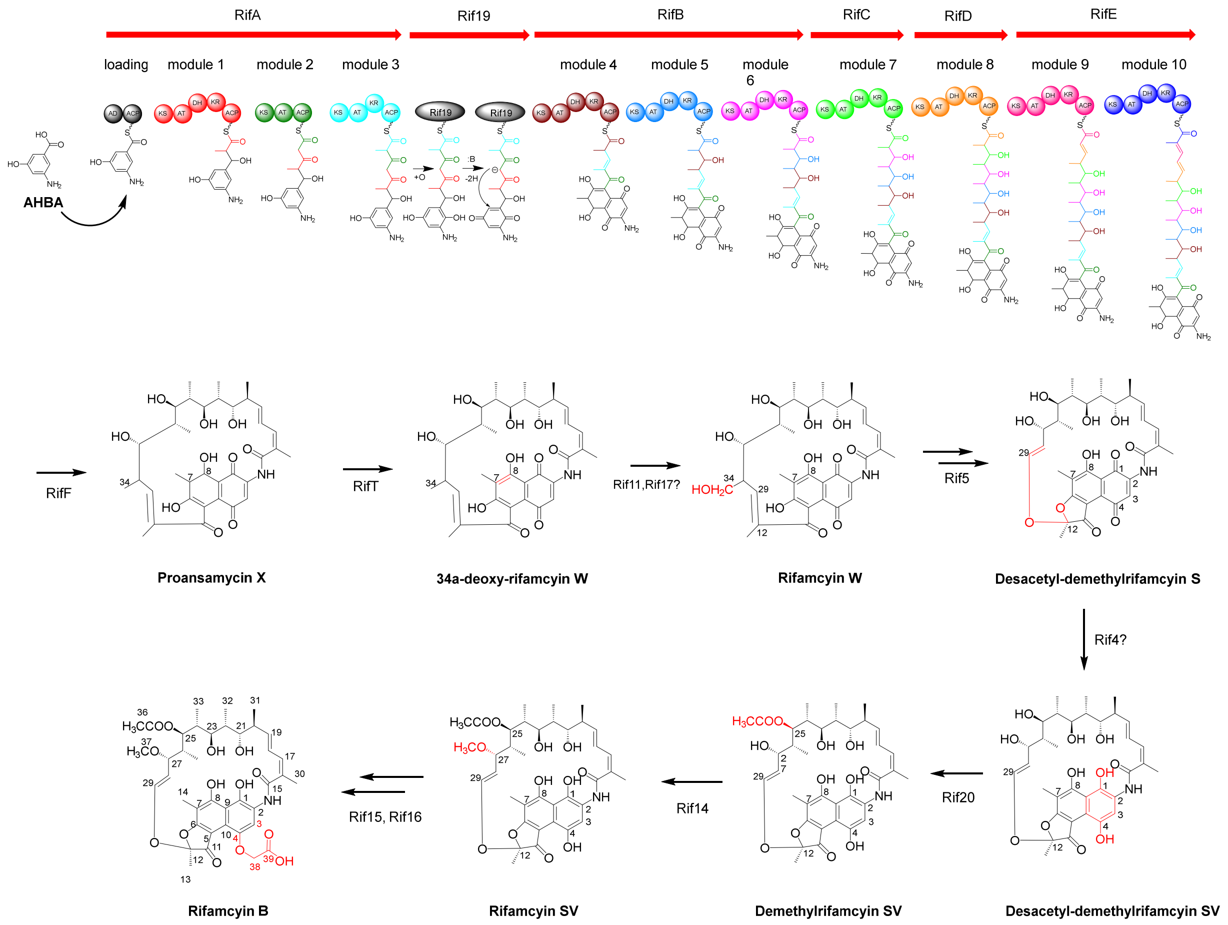

Supplementary Figure 1 The putative rifamycin biosynthetic pathway.

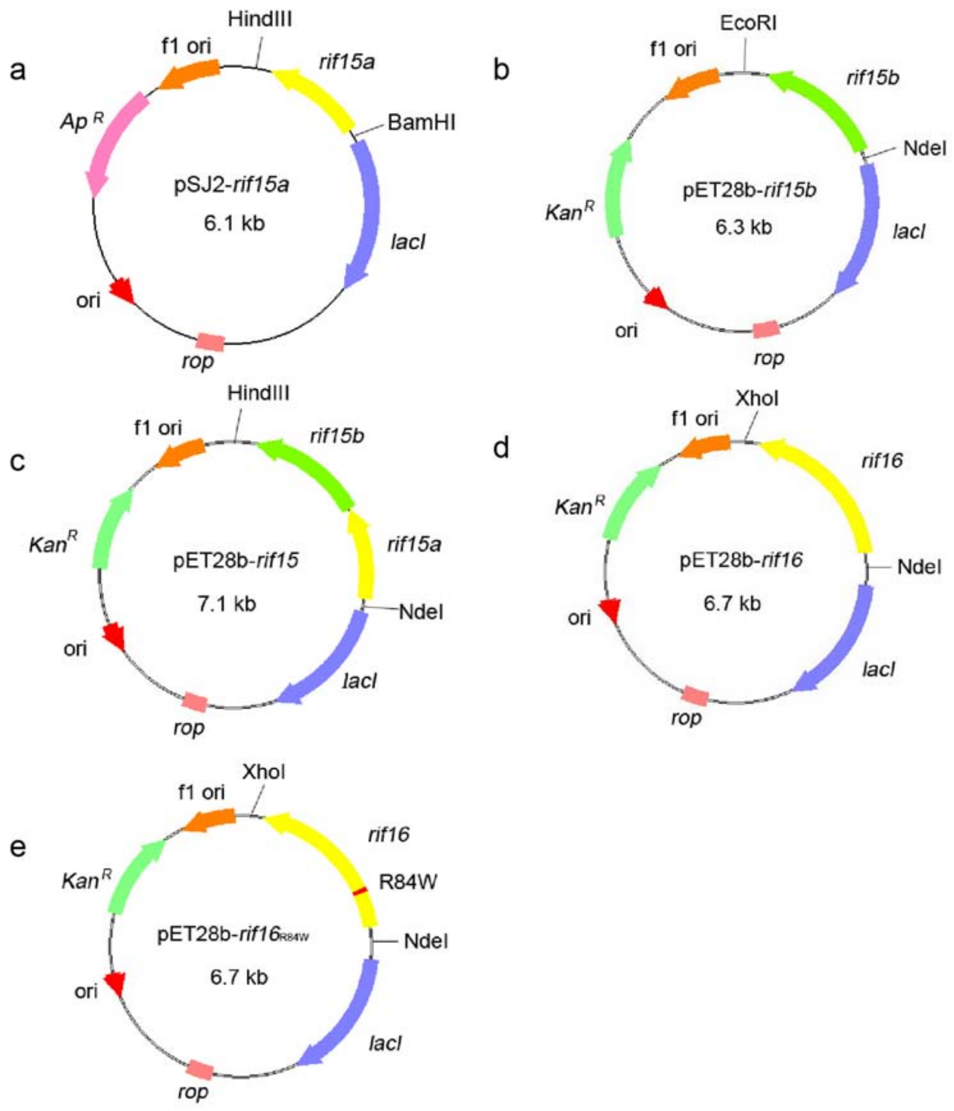

Supplementary Figure 2 Plasmid maps. **a** pSJ2-*rif15a*, **b** pET28b-*rif15b*, **c** pET28b-*rif15*, **d** pET28b-*rif16*, and **e** pET28b-*rif16*$_{\mathrm{R84W}}$.

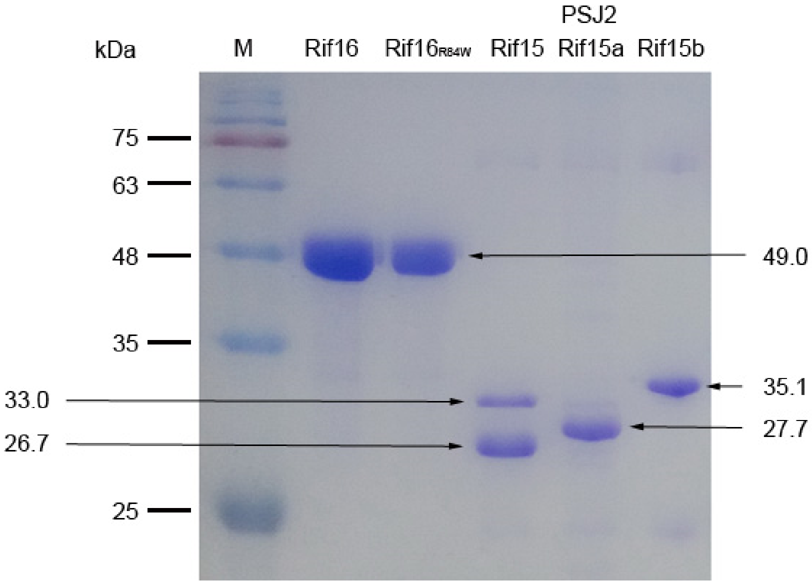

Supplementary Figure 3 SDS-PAGE analysis of purified Rif16, Rif16$_{R84W}$, Rif15, Rif15a, and Rif15b. The calculated molecular masses in kDa are shown by arrows.

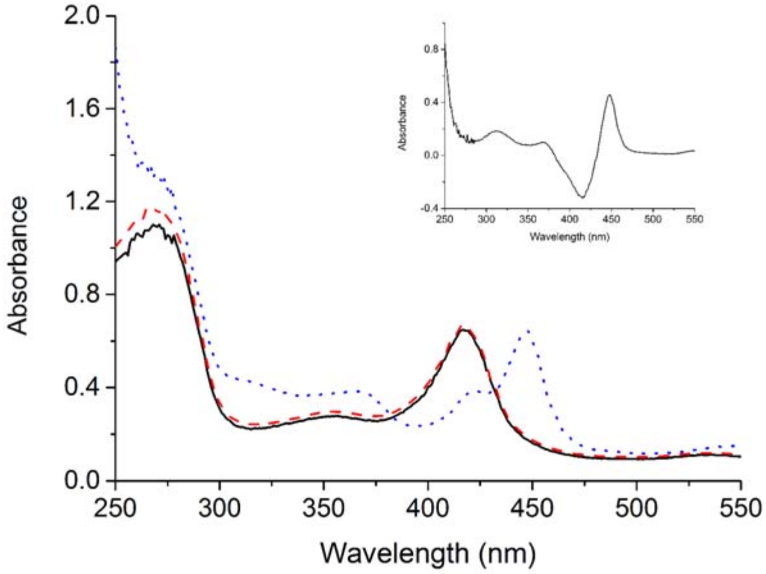

Supplementary Figure 4 The UV-visible absorption spectra of purified Rif16. Ferric form (black solid line), CO-saturated form (red dash line), sodium dithionite reduced and CO-bound form (blue dot line). The CO-bound reduced difference spectrum is shown in inset. This spectrum was also used to determine the concentration of functional P450 enzyme using the extinction coefficient of 91,000 $M^{-1}\cdot cm^{-1}$ [1].

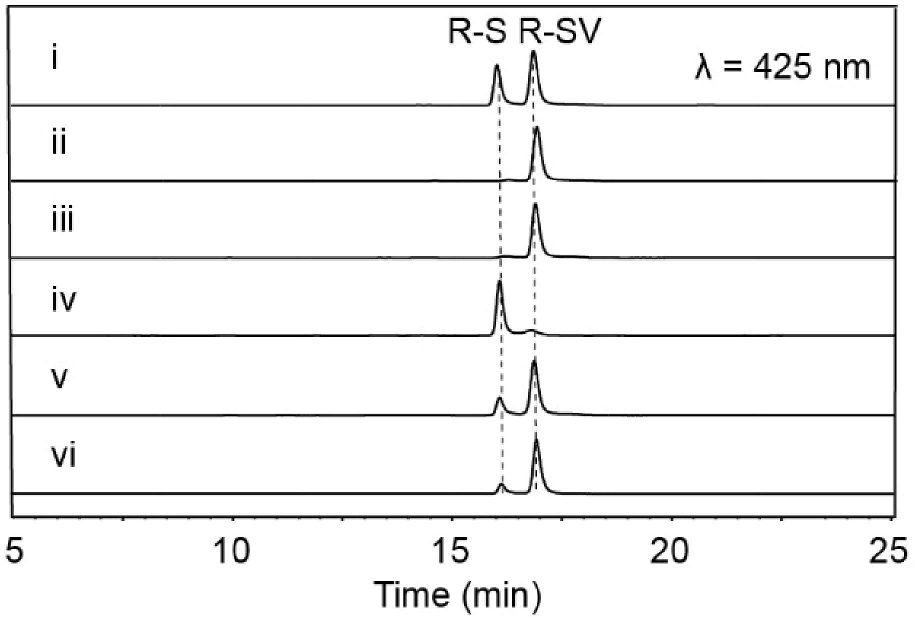

Supplementary Figure 5 The inter-conversion of R-S and R-SV. (i), R-S and R-SV standards; (ii) 200 μM R-SV with 2 μM Rif16 in the presence of 20 μM *se*Fdx, 10 μM *se*FdR and 1 mM NADPH, 28 ºC for 1 h; (iii) 200 μM R-S mixed with *se*Fdx, *se*FdR and NADPH, 28 ºC for 1 h; (iv) 200 μM R-S mixed with *se*Fdx and *se*FdR, 28 ºC for 1 h; (v) 200 μM R-S mixed with NADPH, 28 ºC for 1 h; (vi) 200 μM R-SV incubated in reaction buffer, at 28 ºC for 16 h. All the reactions were quenched by adding the same volume of methanol.

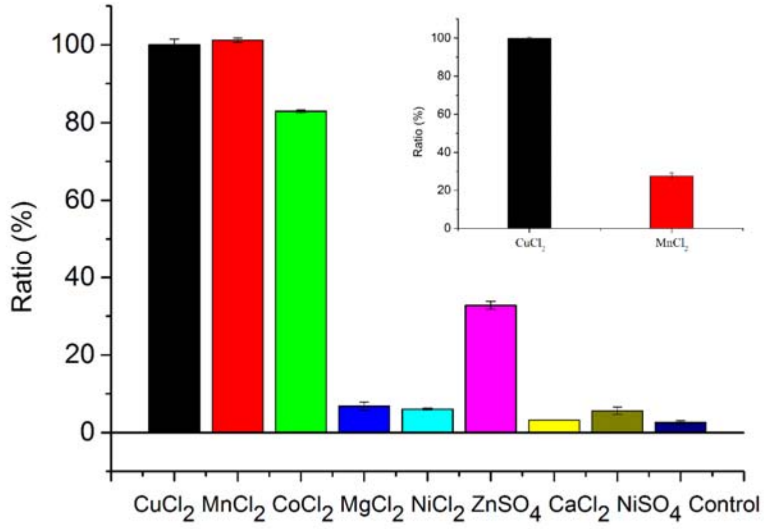

Supplementary Figure 6 The spontaneous oxidation of R-SV to R-S in the presence of different divalent metal ions. In a standard reaction, R-SV (200 μM) was mixed with a certain divalent metal salt (2.5 mM) in 20 mM Tris-HCl buffer (pH 7.4), 28 ºC for 1 h. The control reaction contained no added metal ions. The oxidation efficiencies are shown in relative conversion ratios. The inset shows the two reactions for 1 min in the presence of 2.5 mM Cu^{2+} or Mn^{2+}. All the data are means ± s.d. (n=3).

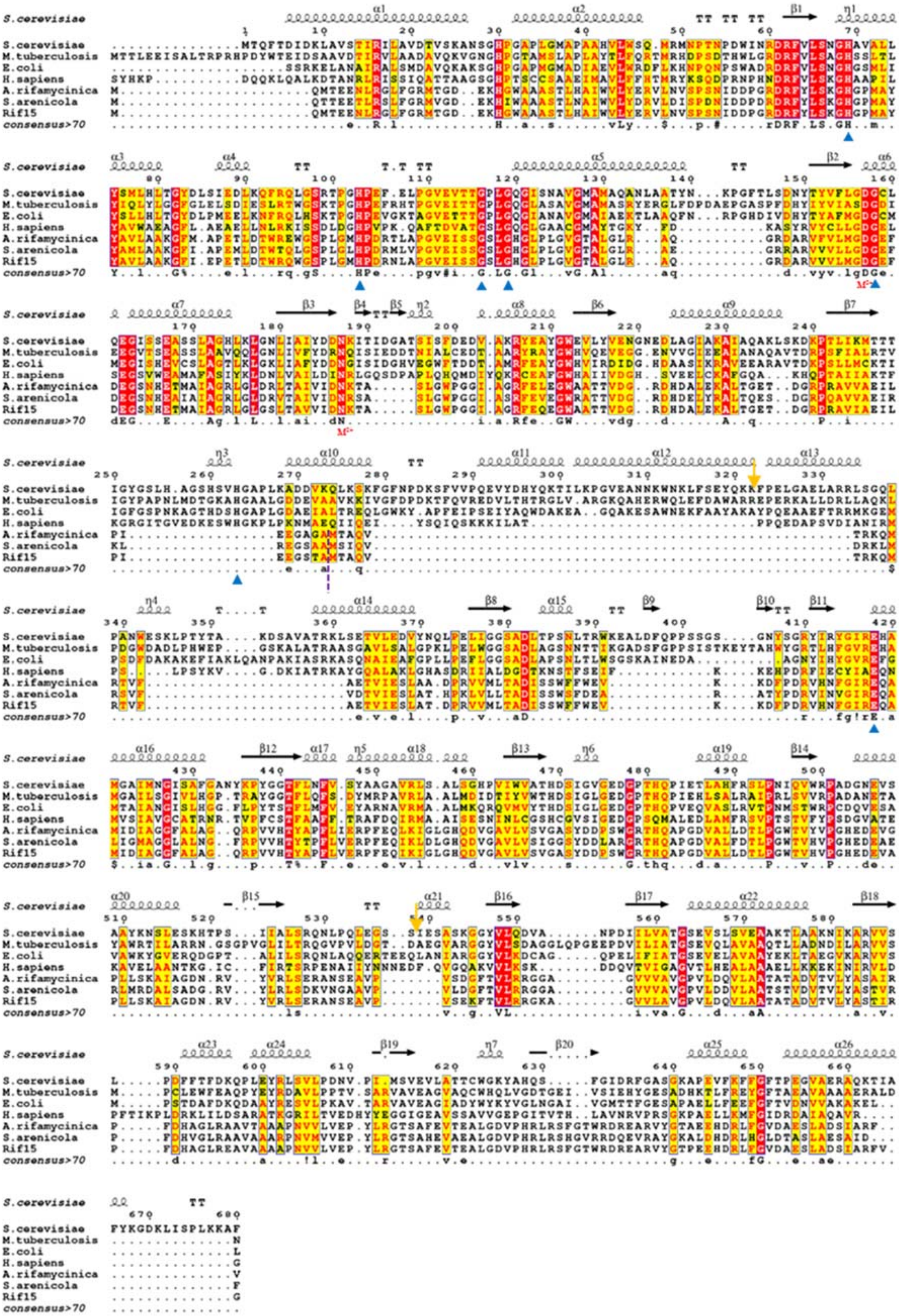
S.cerevisiae
M.tuberculosis
E.coli
H.sapiens
A.rifamycinica
S.arenicola
Rif15
consensus>70

Supplementary Figure 7 Protein sequence alignment of Rif15 and six other transketolases. The transketolases are from *Sacchromyces cerevisiae*, *Mycobacterium tuberculosis*, *Escherichia coli*, *Homo sapinens*, *Amycolatopsis rifamycinica* (rifamycin producer), and *Salinispora arenicola* (rifamycin producer). Sequence analysis was performed using Expresso through the T-COFFEE online service, and the figure was prepared by ESPript 3.0 [2,3]. The secondary structures of the structurally characterized transketolase from *S. cerevisiae* are shown on the top of sequences. The η symbol represents a 3_{10}-helix. α-Helices and β-strands are indicated as helices and black arrows, respectively. The three domains of *S. cerevisiae* transketolase apoprotein including PP domain (residues 3-322), Pyr domain (residues 323-538) and C-terminal domain (residues 539-680) are divided by yellow arrows. The transketolases from three rifamycin producers are composed of two subunits, while the rest transketolases are single polypeptides. For the purpose of sequence alignment, the two subunits are artificially connected into one protein, and the purple dash line points out the start of the second subunit. The residues highlighted in red and yellow are amino acids that are mutually identical and similar, respectively. The blue triangles denote the residues that interact with ThDP. The symbols of M^{2+} in red indicate the residues that contact the divalent metal ion.

a

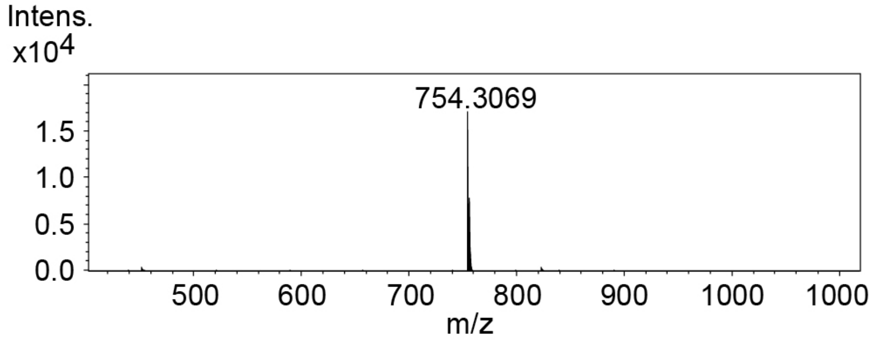

b

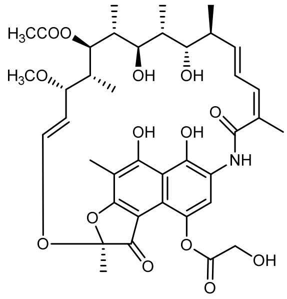

Rifamycin L (R-L)

Supplementary Figure 8 The high resolution mass spectrum of R-L. **a** The high resolution mass spectrum (negative ion mode) of R-L. **b** The chemical structure of R-L.

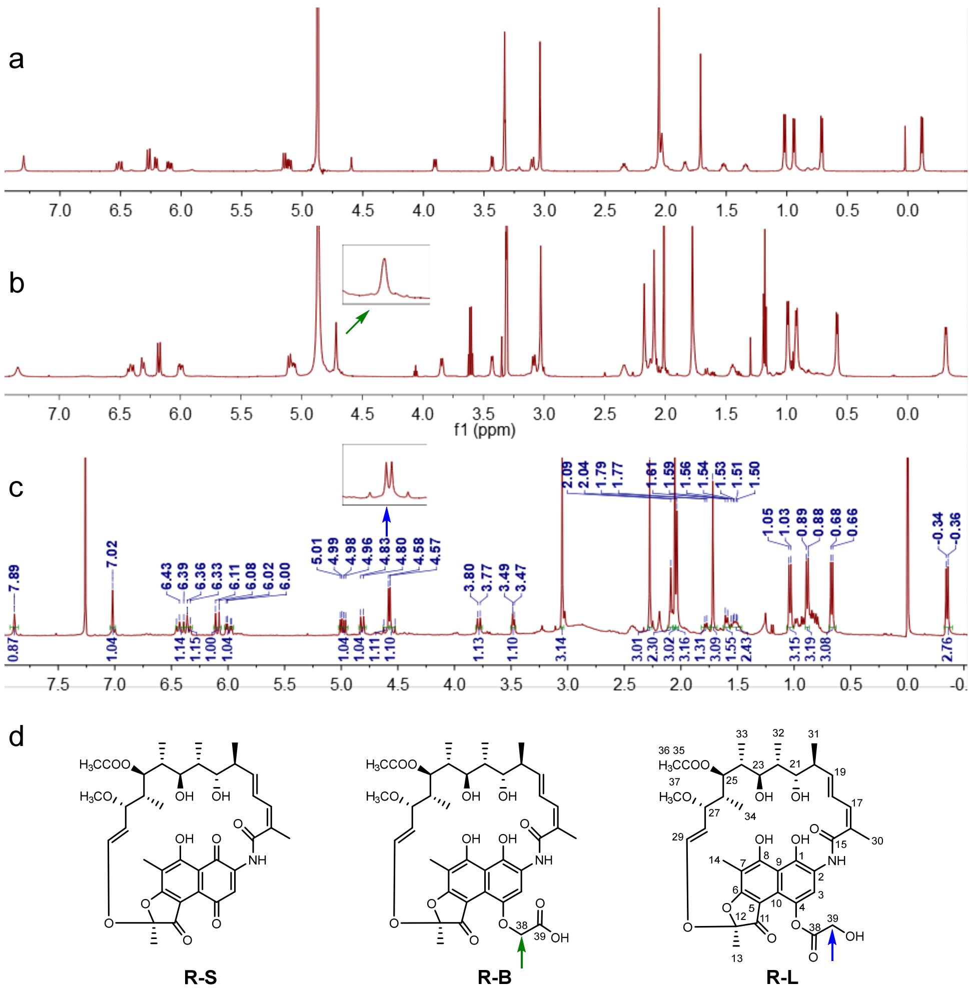

Supplementary Figure 9 ^{1}H NMR spectra of R-S, R-B, and R-L. **a** The ^{1}H NMR spectrum of R-S (in CD_3OD, 600 MHz). **b** The ^{1}H NMR spectrum of R-B (in CD_3OD, 600 MHz). **c** The ^{1}H NMR spectrum of R-L (in $CDCl_3$, 500 MHz). **d** Chemical structures of R-S, R-B and R-L. The blue arrow indicates the new set of CH_2-39 proton signals of R-L, which are distinct to that of R-B (the green arrow).

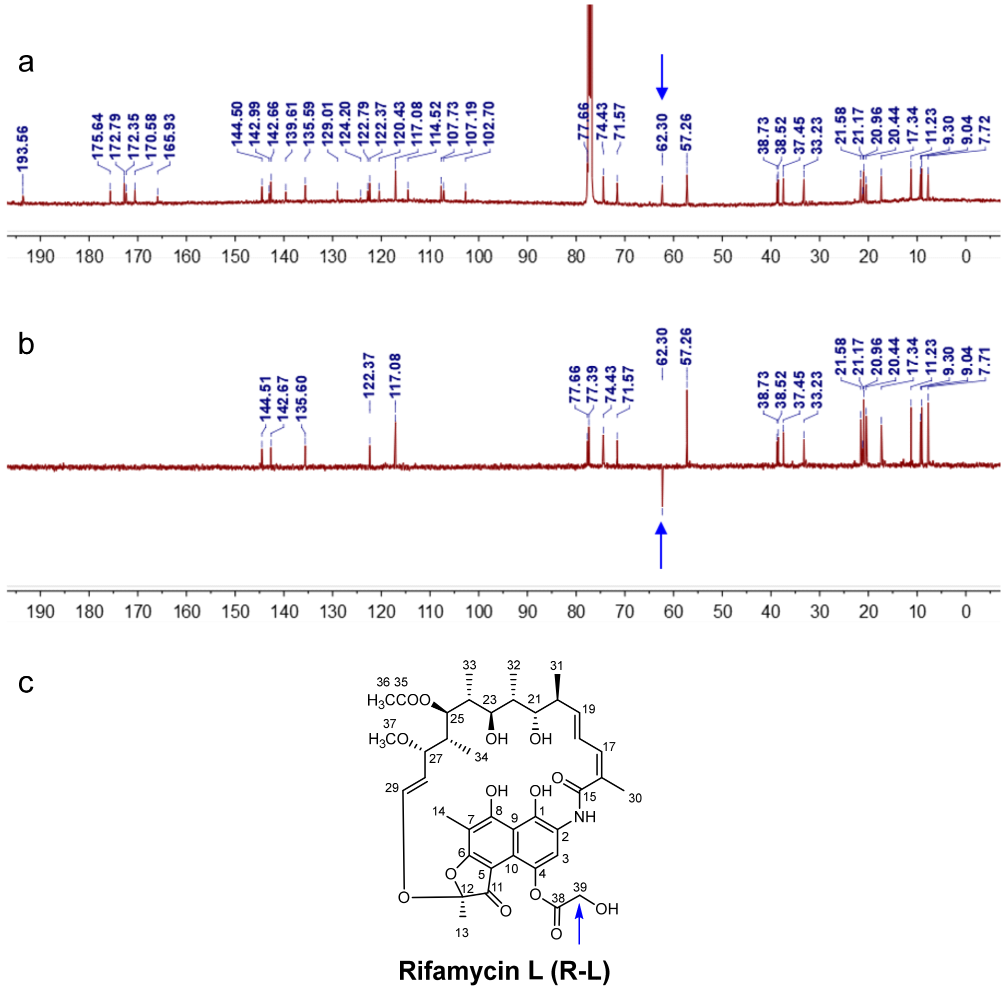

Supplementary Figure 10 ^{13}C NMR and DEPT135 spectra of R-L. **a** The ^{13}C NMR spectrum of R-L in $CDCl_3$ (125 MHz). **b** The DEPT135 spectrum of R-L in $CDCl_3$ (125 MHz). **c** The chemical structure of R-L. The arrows indicate the CH_2-39 carbon signals of R-L.

a

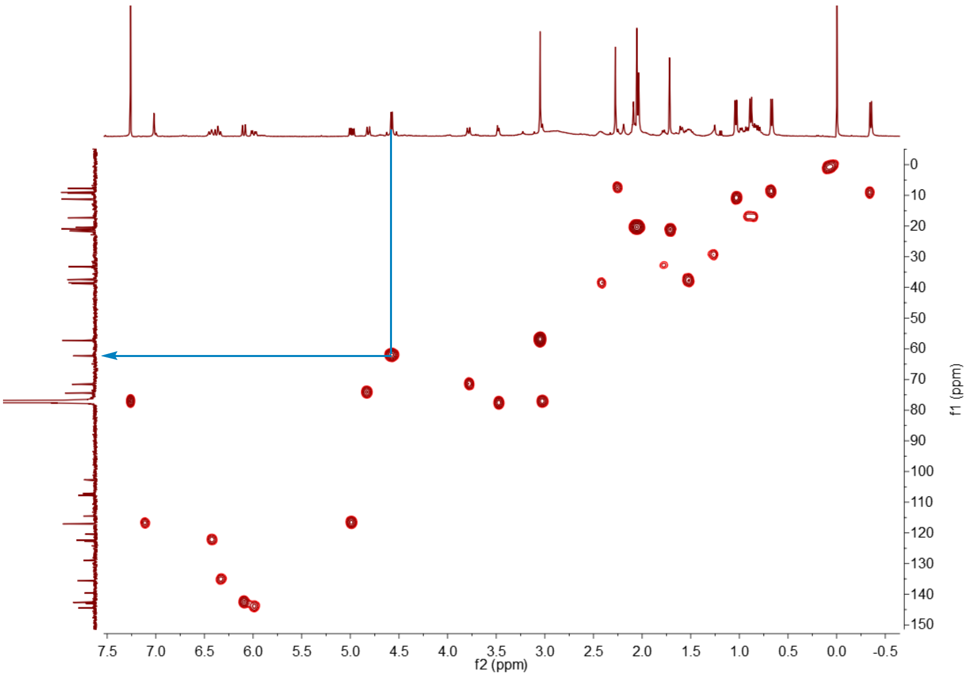

b

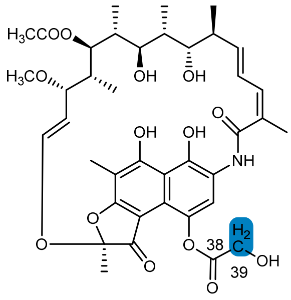

Rifamycin L (R-L)

Supplementary Figure 11 HSQC spectrum of R-L. **a** HSQC spectrum of R-L in $CDCl_3$. The arrow indicates the ^{1}H-^{13}C HSQC correlation of CH_2-39. **b** The chemical structure of R-L.

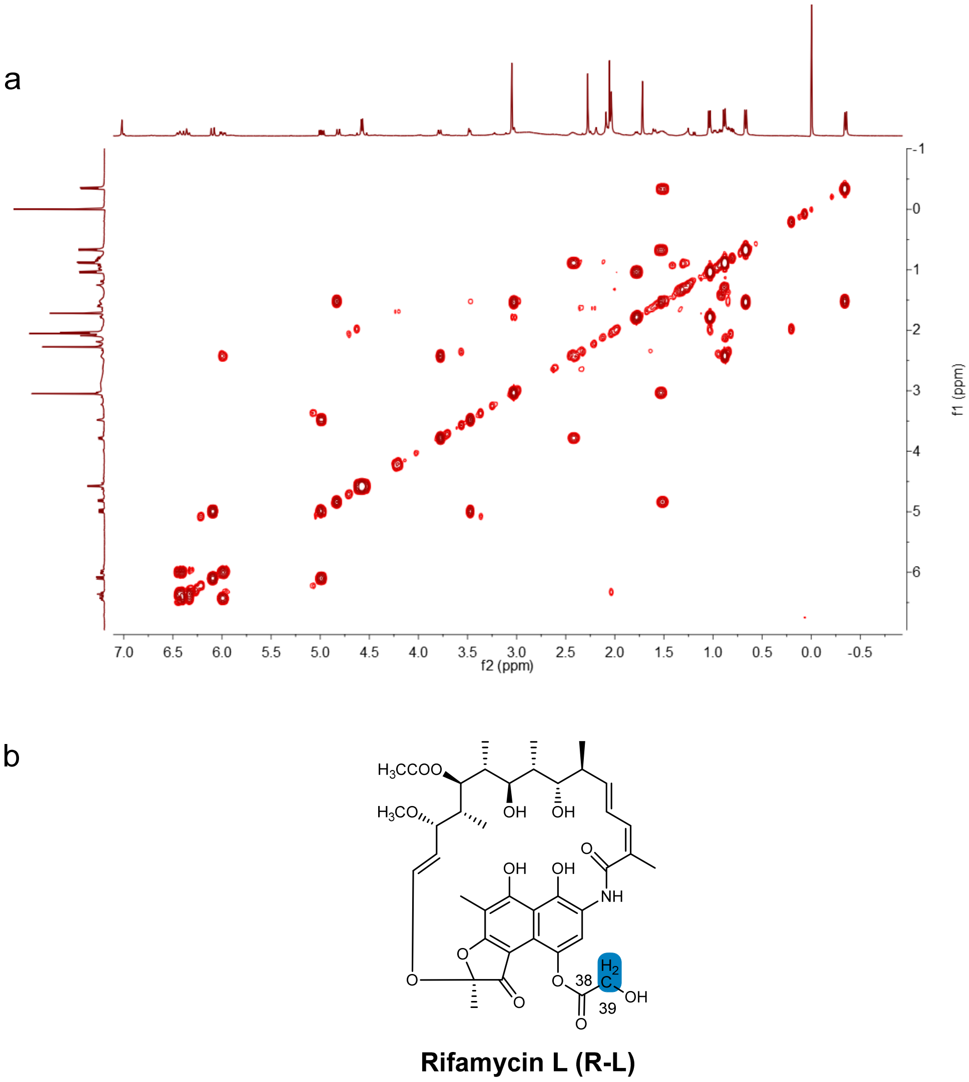

Supplementary Figure 12 ^1H-^1H COSY spectrum of R-L. **a** ^1H-^1H COSY spectrum of R-L in $CDCl_3$. No ^1H-^1H COSY correlation could be observed for H_2-39 except for their geminal coupling. **b** The chemical structure of R-L.

a

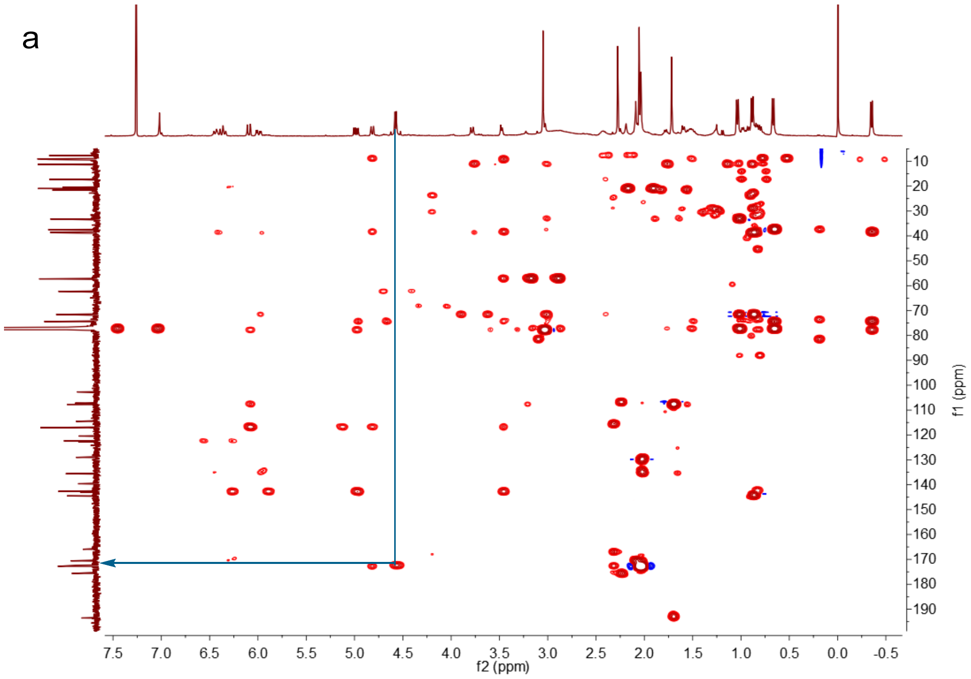

b

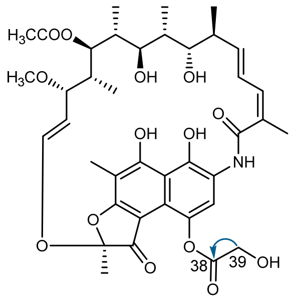

Rifamycin L (R-L)

Supplementary Figure 13 HMBC spectrum of R-L. **a** HMBC spectrum of R-L in $CDCl_3$. The arrow indicates the ^{1}H-^{13}C HMBC correlation from H_2-39 to C-38. **b** The chemical structure of R-L.

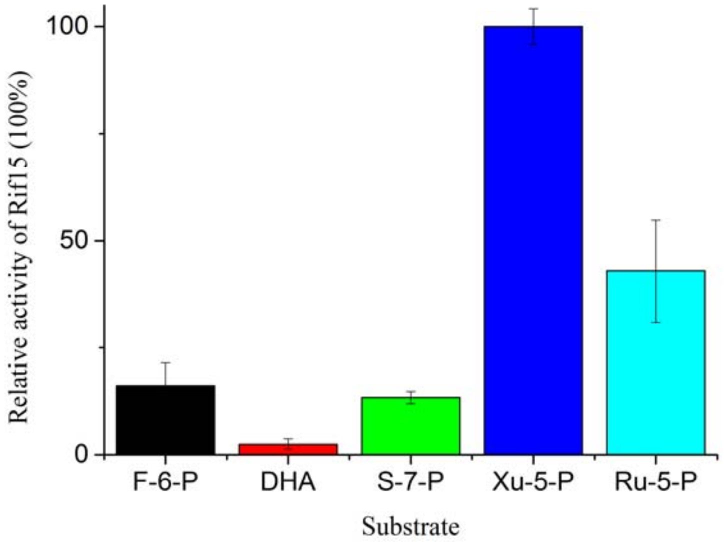

Supplementary Figure 14 The activity of the transketolase Rif15 with different C_2 donors. All the data are means ± s.d. (*n*=3). (fructose-6-phosphate, F-6-P; dihydroxyacetone, DHA; sedoheptulose-7-phosphate, S-7-P; xylulose-5-phosphate, Xu-5-P; ribulose-5-phosphate, Ru-5-P)

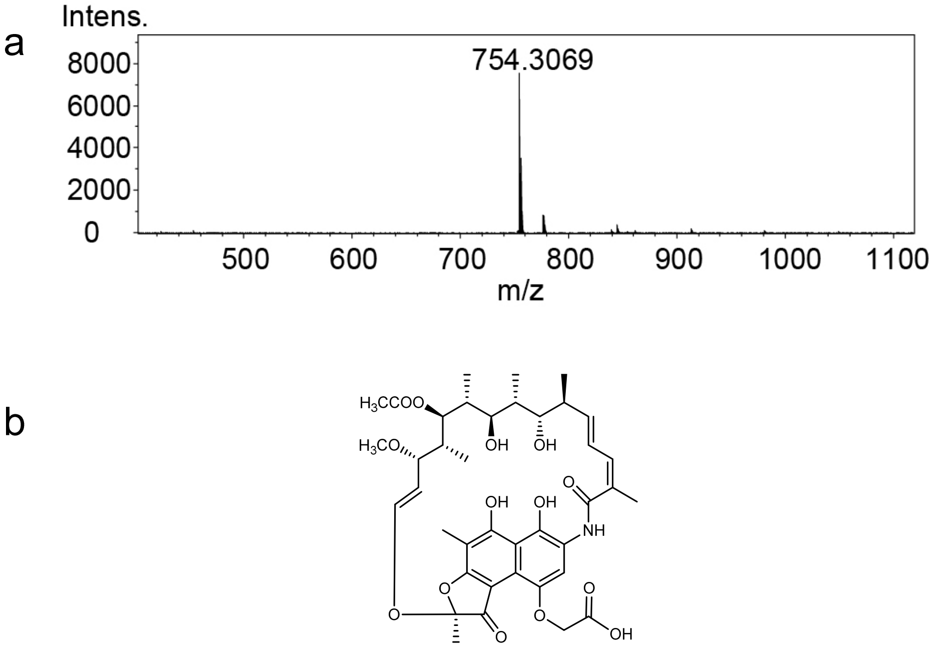

Rifamycin B (R-B)

Supplementary Figure 15 The high resolution mass spectrum of biosynthesized R-B. **a** The high resolution mass spectrum (negative ion mode) of biosynthesized R-B. **b** The chemical structure of R-B.

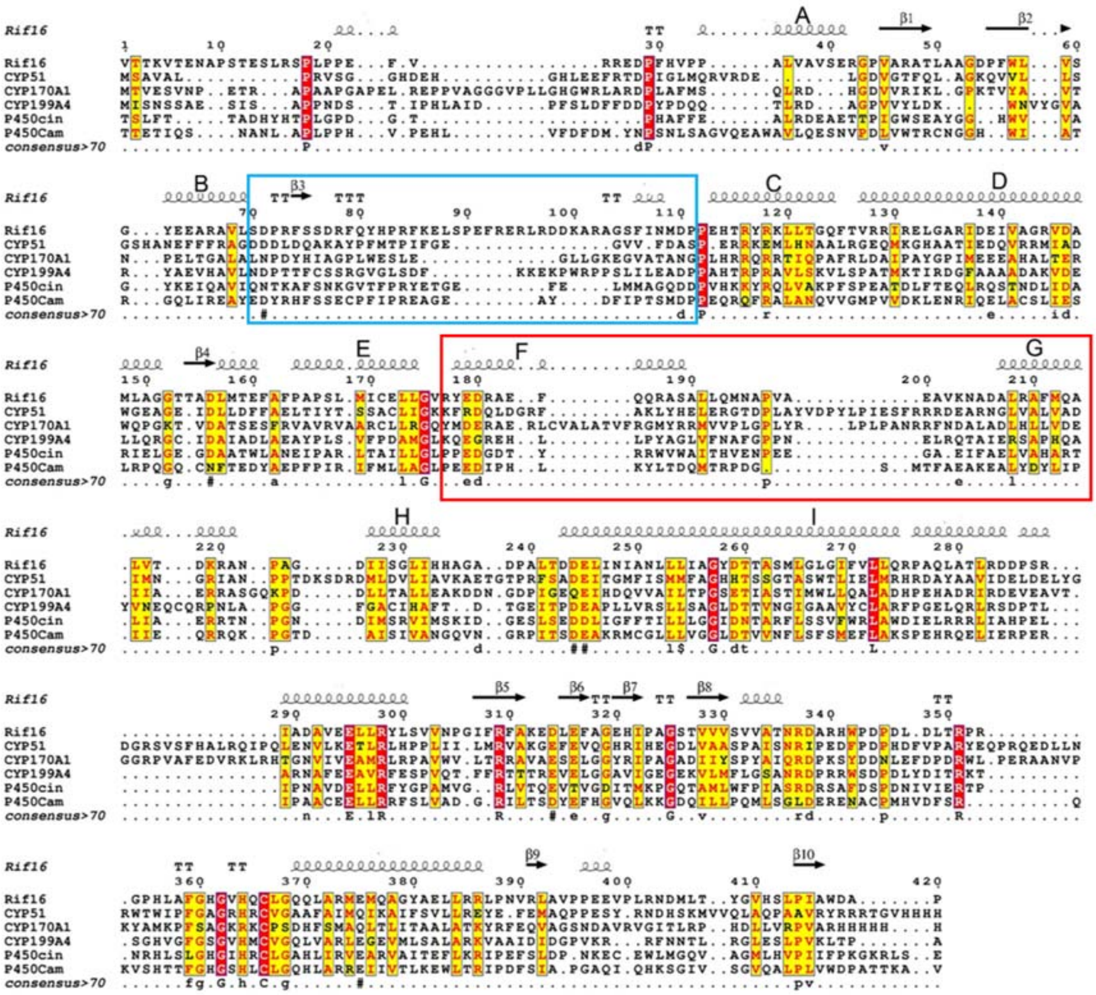

Supplementary Figure 16 Multiple protein sequence alignment between Rif16 and other P450 enzymes with their substrates different in size and shape. The substrates of Rif16, CYP51, CYP170A1, CYP199A4, P450cin, and P450cam are rifamycin L (*m.w.* 755.8), 4,4'-dihydroxybenzophenone (*m.w.* 214.2)[4], *epi*-isozizaene (*m.w.* 204.4)[5], 4-methoxybenzoic acid (*m.w.* 152.15)[6], 1,8-cineole (*m.w.* 154.2)[7], and camphor (*m.w.* 152.2)[8], respectively. Sequence analysis was performed using Expresso through the T-COFFEE online service, and the figure was prepared using ESPript 3.0 [2,3]. The secondary structure assignment and residue numbering are based on the sequence of Rif16. The BB' loop-B' helix-B'C loop region and the F helix-FG loop-G helix region are boxed in blue and red rectangles, respectively.

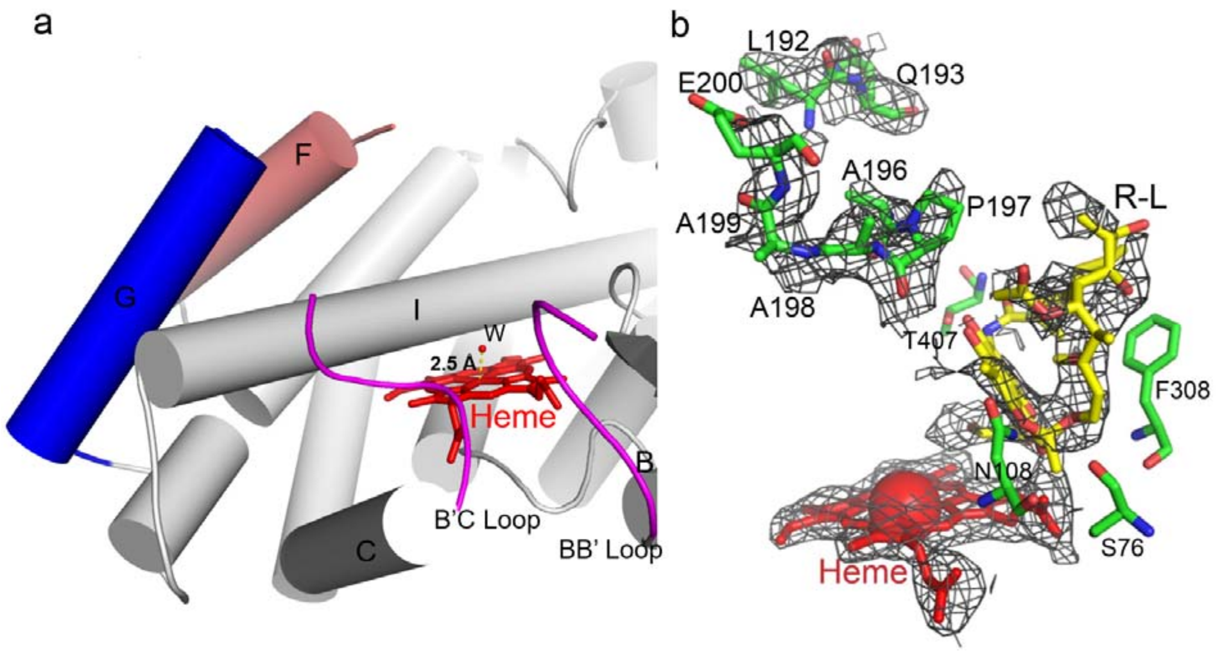

Supplementary Figure 17 Structures of Rif16. **a** Substrate-free Rif16. The axial water ligand is shown as sphere in red. The distance (in angstroms) is indicated by the dashed yellow line. The heme group is shown as a stick in red. **b** The electron-density map of the complex structure. The substrate R-L and heme are shown as sticks in yellow and red, respectively, with the heme iron depicted as a sphere. Key residues that are important for substrate binding are colored in green. The 2Fo-Fc density maps of heme and substrate are contoured at 1.0 σ and 0.8 σ, respectively. The density map of the residues in the ordered F/G loop upon R-L binding is contoured at 1.0 σ.

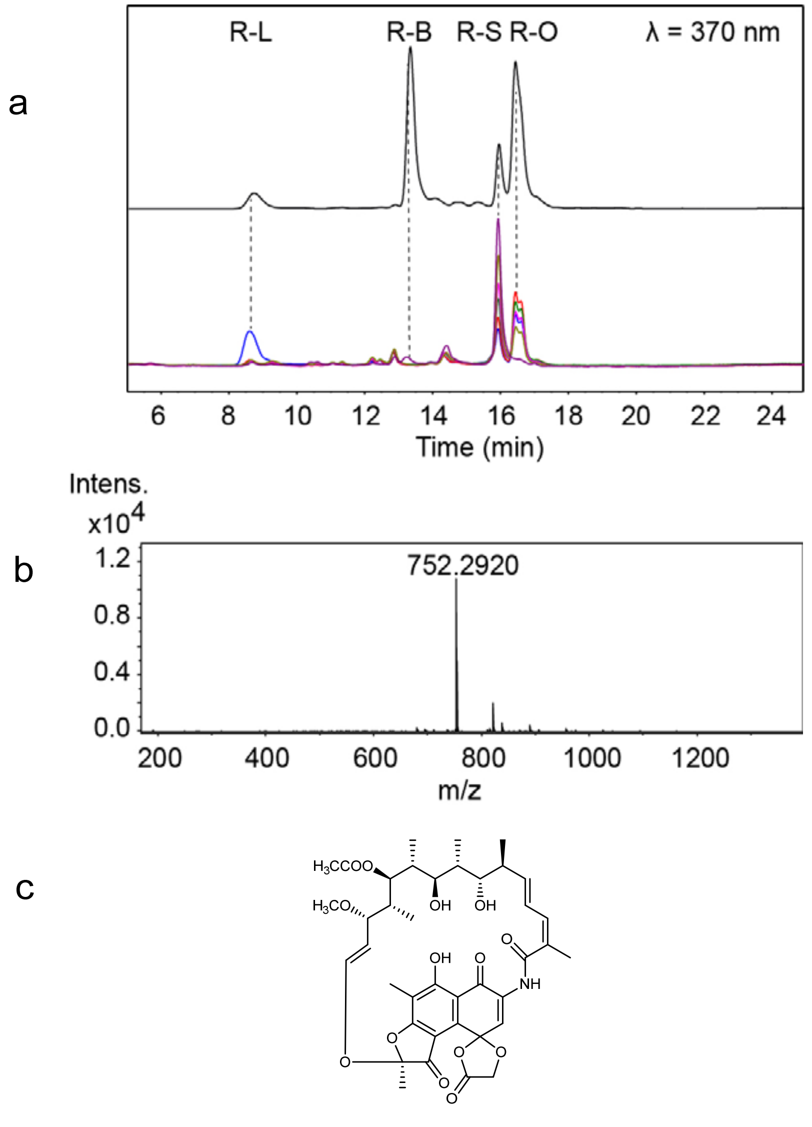

Rifamycin O (R-O)

Supplementary Figure 18 HPLC-HRMS analysis of the transient intermediate (R-O) between R-L and R-B. **a** The time course of Rif16 reactions with R-L in the presence of H_2O_2. All reactions were performed in 200 µL of reaction buffer containing 2 µM Rif16, 200 µM rifamycin L, and 20 mM H_2O_2 at 28 ºC for the indicated time period, and quenched by adding the same volumes of methanol. Black line: The mixed R-L, R-B, R-S and R-O standards; Blue line: 2.5 min reaction; Red line: 5 min reaction; Green line: 10 min reaction; Magenta line: 15 min reaction; Yellow green line: 30 min reaction; Purple line: 60 min reaction. **b** The high resolution mass spectrum (negative ion mode) of R-O. **c** The chemical structure of R-O.

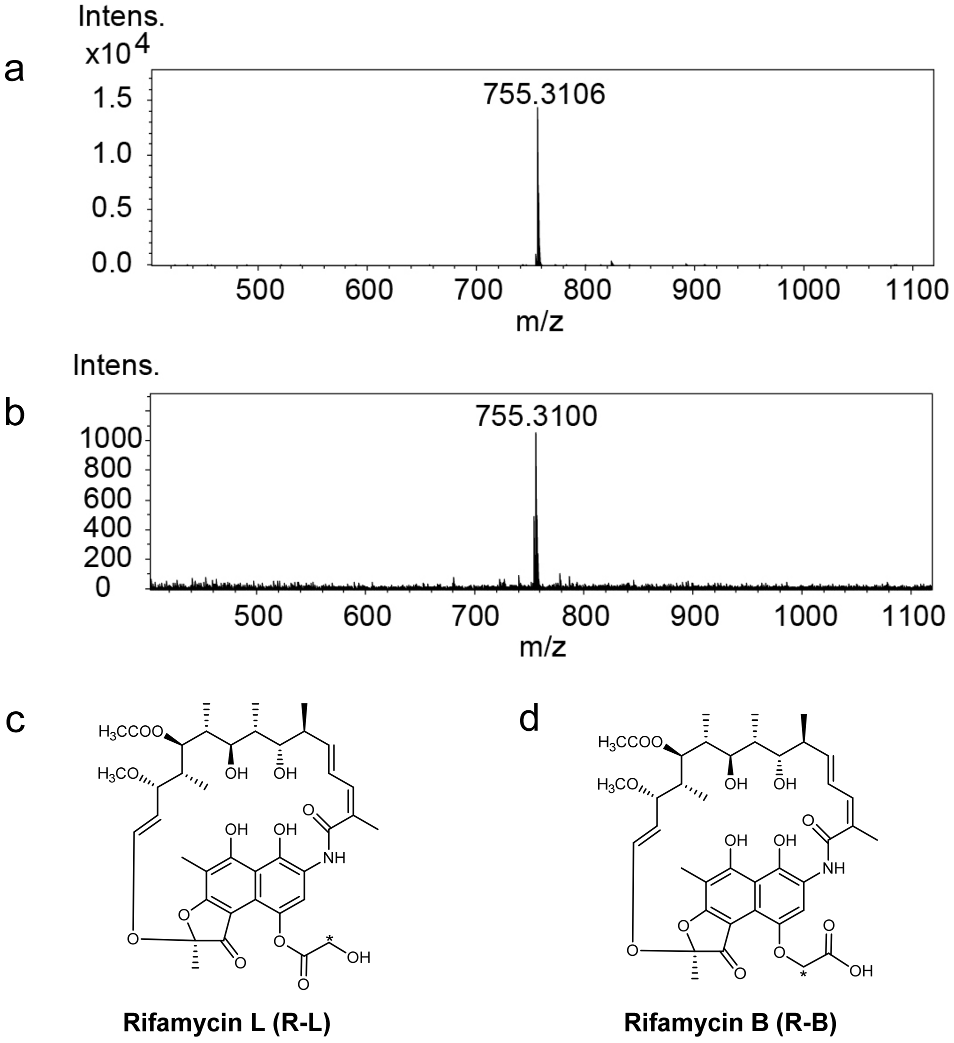

Supplementary Figure 19 The high resolution mass spectra of the ^{13}C labeled R-L and R-B. **a** The high resolution mass spectrum (negative ion mode) of the ^{13}C labeled R-L. **b** The high resolution mass spectrum (negative ion mode) of the ^{13}C labeled R-B. The asterisks represent the carbon atoms labelled by ^{13}C. **c** The chemical structure of R-L. **d** The chemical structure of R-B.

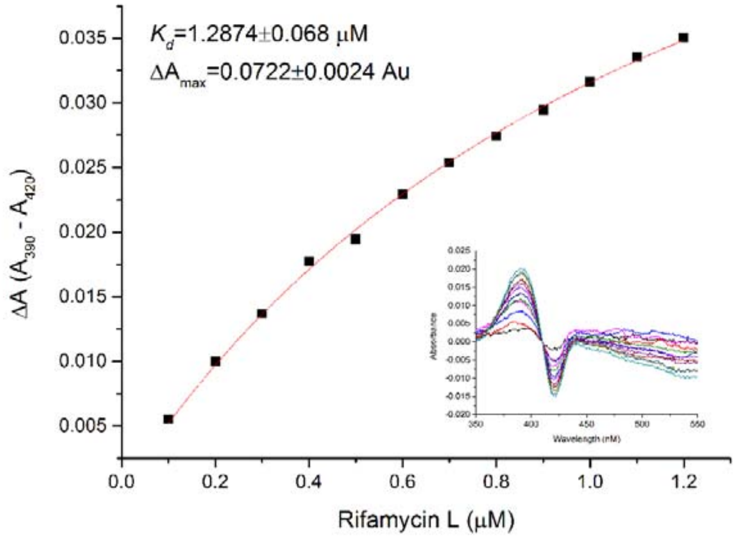

Supplementary Figure 20 The substrate binding curve R-L toward Rif16. The inset shows the recorded Type I binding spectra. The concentration of Rif16 is 1 µM.

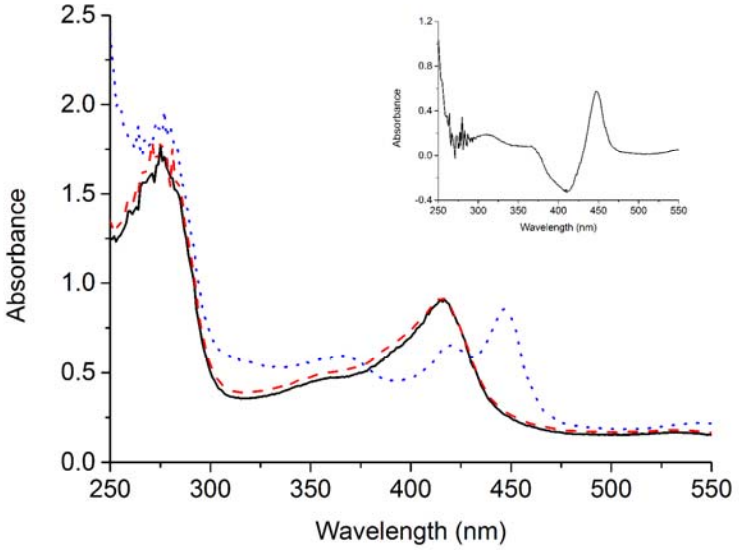

Supplementary Figure 21 UV-visible absorption spectra of purified $\mathrm{Rif16}_{\mathrm{R84W}}$. Ferric form (black solid line), CO-saturated form (red dash line), sodium dithionite reduced and CO-bound form (blue dot line). The CO-bound reduced difference spectrum is shown in inset. This spectrum was also used to determine the concentration of functional P450 enzyme using the extinction coefficient of 91,000 $M^{-1} \cdot cm^{-1}$ [1].

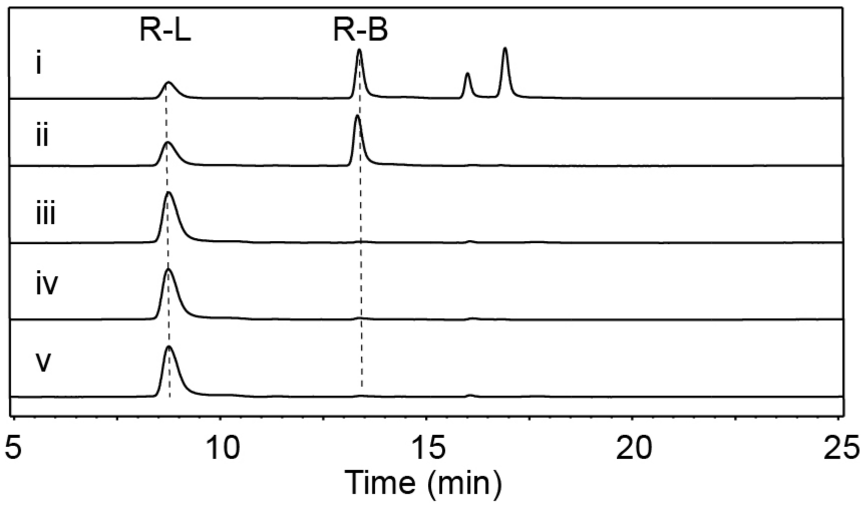

Supplementary Figure 22 HPLC analysis of the reactions catalyzed by Rif16$_{R84W}$. (i), The mixed R-L, R-B, R-S, and R-SV standards; (ii), R-L with Rif16 in the presence of *se*Fdx, *se*FdR, and NADPH; (iii), the negative control of (ii) with the omission of NADPH; (iv), R-L with Rif16$_{R84W}$ in the presence of *se*Fdx, *se*FdR, and NADPH; (v), the negative control of (iv) with the omission of NADPH.

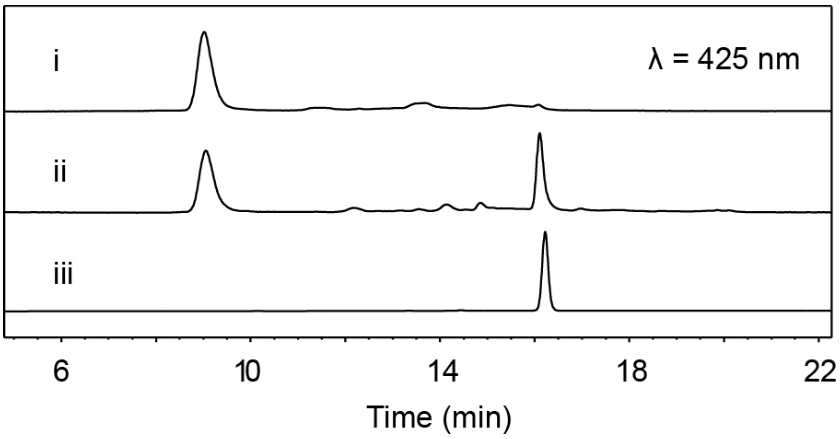

Supplementary Figure 23 HPLC analysis of R-L degradation. (i), R-L newly prepared. (ii), R-L incubated in reaction buffer (vol:vol = 1:99), at 37 ºC for 1 day. (iii), R-S authentic standard.

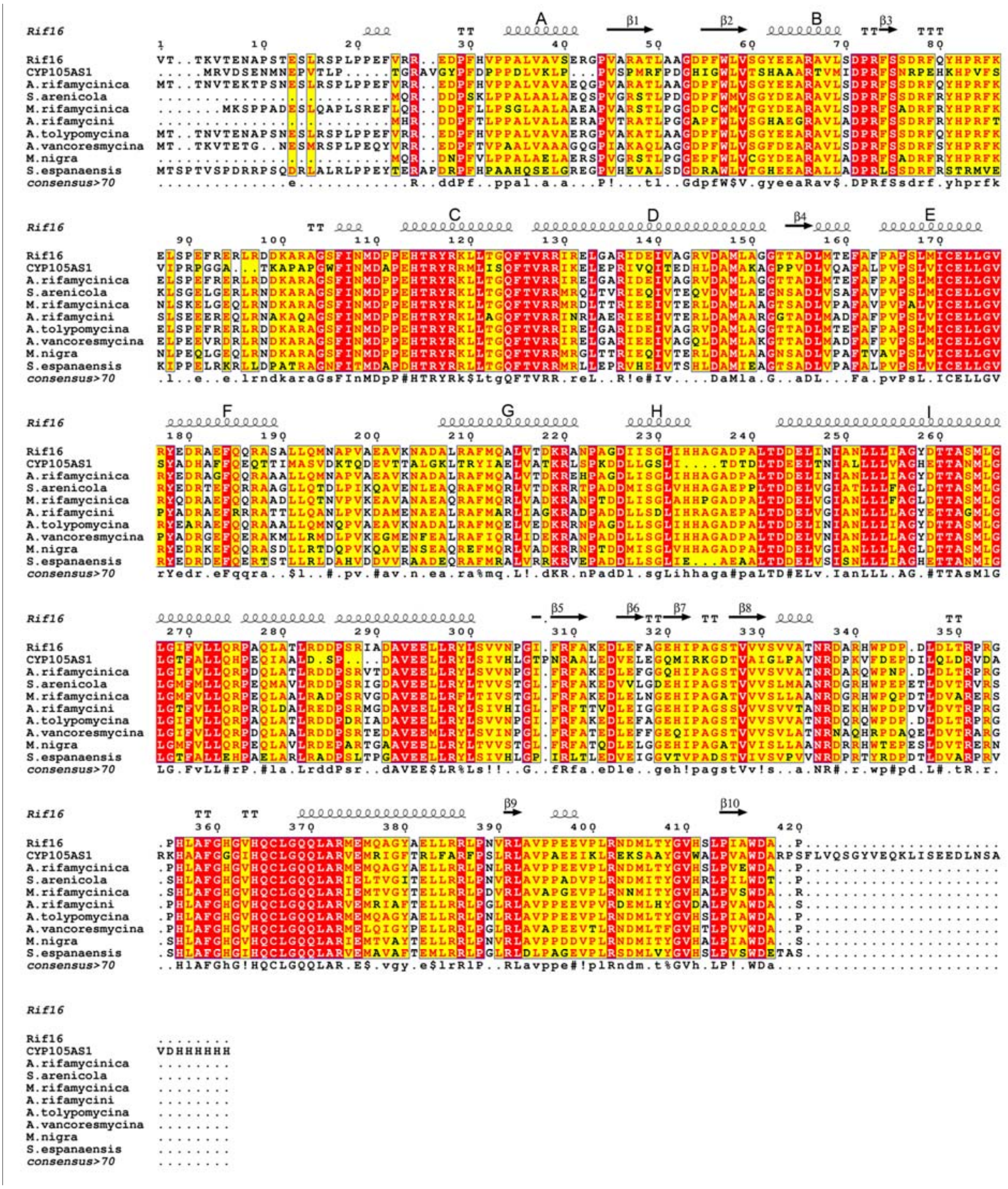

Supplementary Figure 24 Protein sequence alignment of Rif16 and a select number of its analogous P450 enzymes, which are from *Amycolatopsis orientalis* (CYP105AS1, the closest structurally characterized Rif16 homologue), *Amycolatopsis rifamycinica*, *Salinispora arenicola*, *Micromonospora rifamycinica*, *Actinomadura rifamycini*, *Amycolatopsis tolypomycina*,

Amycolatopsis vancoresmycina, *Micromonospora nigra*, and *Saccharothrix espanaensis*, respectively (see Supplementary Table 3). Sequence analysis was performed using Expresso through the T-COFFEE online service, and the figure was prepared by ESPript 3.0[2,3]. The secondary structure assignment and residue numbering are based on the sequence of Rif16. The capital letters and helices on the top of sequences represent α-helices, and the β-strands are indicated as black arrows.

Supplementary Tables

Supplementary Table 1 The nucleotide sequences of *rif15* and *rif16*. Blue letters and green letters indicate the sequences of *rif15a* and *rif15b*, respectively. Red letters denote the overlapped *rif15a* stop codon and *rif15b* start codon. Black letters are the sequence of *rif16*. The cytimidine highlighted in yellow is mutated to a thymine in *A. mediterranei* U32, leading to the null mutant Rif16$_{R84W}$.

***rif15* (AMED_0651 and AMED_0652)**
ATGCAGATGACCGAAGAGAACCTCCGCGGCCTGTTCGGCCGGATGACGGGGACGAGAAGCACGGCTG<br>GGCCGCGGCGTCGACATTGCACGCGATCTGGGTGCTCTACGAACGCGTGCTCAACGTGTCGCCGTCGAA<br>CATCGACGACCCCGGCCGGGACCGGTTCTACCTCTCCAAGGGACACGGCCCGATGGCCTACTACGCGGT<br>GCTCGCCGCGAAGGGCTTCATCGAGCCGGAAACGCTGGACACCTGGCGGCAGTGGGGTTCGCCGCTGG<br>GCATGCACCCGGACCGCAACCTGGCGCCCGGCGTGGAGATCAGCAGCGGCTCCCTCGGCCACGGGCTCC<br>CGCTCGGCGTCGGCACCGCGCTCGGGCTGCGCGCCCAGGGCCGCGACGCCCGCGTGGTCGTCCTGATG<br>GGCGACGGCGAGTTCGACGAGGGCAGCAACCACGAGACGATGGCGATCGCCGGACGGCTCGGGCTGGG<br>CAGCCTCACCGCGGTCGTCATCGACAACAAGACGGCGAGCCTCGGCTGGCCGGGCGGCATCGCCGGGC<br>GCTTCGAACAGGAGGGCTGGGCCGCCACCACGGTCGACGGCCGCGACCACGACGCGCTGGAGAAGGCG<br>CTGACCGGGGAGACCGACGGGCGGCCGCGCGCGGTCATCGCGGAGATCCTCCCGATCGAGGAAGGGAG<br>CACCGCATGACCGCCCAGGTGACCAGGAAGCAGATGCGGACCGTCTTCGCCGAGACGGTGATCGAGTCG<br>CTGGCCACGGACCCGCGCGTGGTCATGCTGACCGCCGACATCTCGTCGTGGTTCTTCTGGGAGGTCAAG<br>AAGGACTTCCCGGACCGCGTCCACAACTTCGGCATCCGCGAGCAGGCGATGATCGACATCGCCGGCGGC<br>TTCGCGCTGGCCGGCCAGCGGCCGGTGGTGCACACGTACGCGCCGTTCCTGGTCGAGCGGCCGTTCGAG<br>CAGATCAAGATCGGCCTCGGCCACCAGGACGTCGGCGCGGTGCTGGTCAGCGTGGGCGCCTCCTACGAC<br>GACCCGTCGTGGGGCCGCACCCACCAGGCCCCGGGCGACGTGGCGCTGCTGGACACGCTGCCGGGCTG<br>GACGGTGCACGTCCCGGGCCACGAGGACGAGGTCGCGCCCCTGCTGAGCAAGGCCATCGCGGGTGACA<br>ACCGGGTCTACGTCCGGCTGTCCGAACGCGCGAACAGCGAAGCGGTGCCGGTGTCGGAGAAGTTCACGG<br>TGCTGCGCCGGGGCAAGGCGGGCGTGGTGCTCGCGGTCGGCCCGGTGCTGGACCAGGTCCTGGCGGCC<br>ACGGCCACGGCGGACGTGACGGTGCTGTACGCCTCGACGATCCGCCCGTTCGACCACGCGGGCCTCCGG<br>GAGGCGGTGGCCGCGGCGGCCCCGAACGTGGTGCTGGTCGAGCCGTACCTGCGCGGGACGTCGGCGTT<br>CGAGGTGACCGAGGCTCTGGGAGACGTCCCGCACCGCCTGCGCTCGTTCGGAACCTGGCGCGACCGCGA<br>AGCCCGCGTCTACGGAACGCCCGAGGAACACGACCGCCTGTTCGGCGTGGACGCCGAGTCGCTGGCGGA<br>TTCGATCGCCCGCTTCGTCGGCTGA
rif16
GTGACGACCAAAGTGACCGAAAACGCGCCCAGCACCGAATCGCTGCGCTCACCACTCCCTCCGGAGTTC<br>GTCCGGCGCGAAGACCCGTTCCACGTGCCGCCCGCTTTGGTGGCCGTCTCCGAACGGGGTCCCGTCGCC<br>CGGGCGACACTGGCCGCCGGCGACCCGTTCTGGCTCGTGTCCGGGTACGAGGAAGCGCGCGCGGTGCT<br>GTCGGACCCGCGCTTTTCCTCCGACCGGTTCCAGTACCACCCGCGGTTCAAGGAACTGTCGCCCGAATTC<br>CGGGAGCGCCTGCGGGACGACAAAGCGCGCGCGGGGTCGTTCATCAACATGGACCCGCCGGAGCACAC<br>CCGGTACCGGAAACTGCTCACCGGGCAGTTCACCGTCCGGCGGATCCGCGAGCTCGGCGCCCGGATCGA<br>CGAGATCGTCGCCGGCCGGGTGGACGCCATGCTGGCCGGCGGGACCACCGCCGACCTGATGACCGAATT<br>CGCGTTTCCGGCGCCTTCGCTGATGATCTGCGAGCTGCTCGGGGTGCGGTACGAGGACCGGGCGGAGTT<br>CCAGCAGCGCGCGTCGGCCCTGCTGCAGATGAACGCCCCGGTCGCGGAGGCGGTGAAGAACGCCGACG<br>CCCTGCGCGCGTTCATGCAGGCGCTGGTCACGGACAAGCGGGCGAACCCGGCGGGCGACATCATCTCCG<br>GCCTGATCCACCACGCCGGCGCCGACCCCGCGCTCACCGACGACGAGCTGATCAACATCGCCAACCTGC<br>TGCTCATCGCCGGCTACGACACCACGGCGAGCATGCTGGGGCTGGGCATCTTCGTGCTGCTGCAGCGCC<br>CCGCCCAGCTCGCCACGCTGCGCGACGACCCGTCCCGCATCGCCGACGCGGTCGAGGAGCTGCTGCGCT<br>ACCTGTCCGTGGTGAACCCGGGGATCTTCCGCTTCGCCAAGGAGGACCTGGAGTTCGCCGGCGAGCACA<br>TCCCGGCCGGTTCGACGGTGGTGGTCTCCGTGGTGGCGACCAACCGCGACGCGCGGCACTGGCCGGACC<br>CGGACCTCGACCTGACCCGCCCCCGCGGCCCCCACCTGGCGTTCGGCCACGGCGTGCACCAGTGCCTCG<br>GCCAGCAGCTGGCCCGGATGGAGATGCAGGCGGGCTACGCCGAGCTGCTGCGCCGCCTGCCGAACGTTC<br>GCCTGGCGGTGCCGCCGGAGGAGGTTCCGCTGCGCAACGACATGCTGACTTACGGGGTGCACTCGCTTC<br>CGATCGCCTGGGACGCTCCCTAA

Supplementary Table 2 ^{1}H (600 MHz) and ^{13}C (150 MHz) NMR data for R-L and R-B in CD_3OD

	R-L		R-B	
**Position**	***δ*C, Type**	***δ*H, mult., (*J* in Hz)**	***δ*C, Type**	***δ*H, mult., (*J* in Hz)**
1	140.8, C		141.7, C	
2	124.2, C		125.3, C	
3	119.6, CH	7.23, s	109.0, CH	7.34, s
4	144.1, C		144.0, C	
5	103.3, C		103.6, C	
6	176.1, C		172.7, C	
7	107.0, C		111.8, C	
8	167.1, C		167.0, C	
9	115.0, C		115.3, C	
10	121.8, C		119.6, C	
11	193.5, C		197.3, C	
12	108.7, C		109.0, C	
13	21.9, CH_3	1.67, s	22.2, CH_3	1.78, s
14	6.9, CH_3	2.25, s	7.54, CH_3	2.17, s
15	171.7, C		172.7, C	
16	131.1, C		132.1, C	
17	134.5, CH	6.33, d, (11.0)	134.2, CH	6.31, d, (10.7)
18	124.3, CH	6.43, dd, (14.8, 11.6)	125.3, CH	6.41, dd, (15.0, 11.3)
19	141.8, CH	6.00, dd, (15.6, 5.8)	141.7, CH	6.00, dd (15.3, 5.7)
20	39.3, CH	2.36, m	39.7, CH	2.36, m
21	73.1, CH	3.76, d, (8.9)	73.6, CH	3.84, d, (7.6)
22	33.9, CH	1.78, m	34.4, CH	1.77, m
23	77.8, CH	3.12, dd, (10.3, 1.8)	78.1, CH	3.08, brd, (10.1)
24	38.8, CH	1.52, m	39.1, CH	1.44, m
25	79.1, CH	4.66, (overlap)	79.7, CH	4.72 (overlapped)
26	39.2, CH	1.52, m	39.7, CH	1.44, m
27	77.9, CH	3.41, brd, (6.9)	78.6, CH	3.43, brd, (6.1)
28	119.3, CH	4.98, dd, (12.7, 6.9)	119.6, CH	5.07, dd, (12.6, 7.0)
29	143.6, CH	6.14, d, (12.7)	144.0, CH	6.18, d, (12.7)
30	20.8, CH_3	2.07, s	20.8, CH_3	2.09, s
31	17.3, CH_3	0.90, d, (7.0)	17.9, CH_3	0.92, d, (6.6)
32	10.7, CH_3	1.00, d, (7.0)	11.4, CH_3	0.99, d, (6.8)
33	9.5, CH_3	0.70, d, (7.0)	9.4, CH_3	0.58, d, (6.6)
34	9.3, CH_3	-0.43, d, (6.8)	9.4, CH_3	-0.31, d, (6.2)
35	172.5, C		172.7, C	
36	20.4, CH_3	2.02, s	20.8, CH_3	2.01, s
37	56.9, CH_3	3.01, s	57.1, CH_3	3.03, s
38	173.5, C		67.8, C	4.72, s
39	62.3, CH_2	4.63, d (16.3)	172.7, CH_2	
		4.56, d (16.8)		

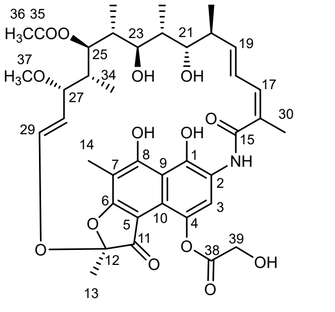

Rifamycin L (R-L)

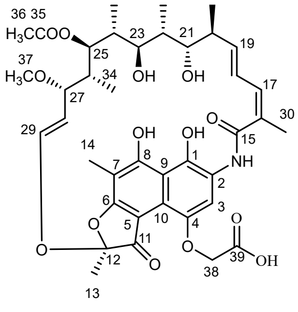

Rifamycin B (R-B)

Supplementary Table 3 Amino acid sequence similarity and identity of Rif15a, Rif15b and Rif16 with other similar proteins. The percentage numbers of similarity and identity are obtained at https://blast.ncbi.nlm.nih.gov/Blast.cgi using the protein sequence of Rif15a, Rif15b and Rif16 as entries. The asterisks indicate the strains that have been discovered to be rifamycin producers. The pound signs represent the strain whose *rif15* and *rif16* counterparts are adjacent to each other on its genome.

**Species**	**Rif15a**		**Rif15b**		**Rif16**	
	Protein ID/ locus_tag	Similarity/ Identity (%)	Protein ID/ locus_tag	Similarity/ Identity (%)	Protein ID/ locus_tag	Similarity/ Identity (%)
Amycolatopsis rifamycinica* #	WP_043781862.1/ DV20_RS18385	96%/ 95%	WP_084093546.1/ DV20_RS18390	97%/ 96%	WP_043781865.1/ DV20_RS18395	97%/ 95%
Salinispora arenicola*	WP_029021589.1/ B162_RS0115765	88%/ 80%	WP_020217895.1/ B162_RS0115760	81%/ 72%	WP_018796309.1/ B162_RS0115840	85%/ 73%
Micromonospora rifamycinica*	WP_067307198.1/ AWV63_RS12215	88%/ 79%	WP_067307195.1/ AWV63_RS12210	82%/ 75%	WP_084261269.1/ AWV63_RS12285	84%/ 72%
Actinomadura rifamycini*	WP_051301103.1/ H505_RS0124100	80%/ 74%	WP_026404027.1/ H505_RS0124105	81%/ 73%	WP_026404031.1/ H505_RS0124125	84%/ 72%
Amycolatopsis tolypomycina	WP_091304035.1/ BLW76_RS01485	96%/ 95%	WP_091304036.1/ BLW76_RS01490	97%/ 94%	WP_091304041.1/ BLW76_RS01515	97%/ 96%
Amycolatopsis vancoresmycina #	WP_003071778.1/ OO60_RS14045	86%/ 80%	WP_003071776.1/ OO60_RS14040	91%/ 87%	WP_003071775.1/ OO60_RS14035	88%/ 78%
Micromonospora nigra	WP_091080831.1/ GA0070616_RS11740	85%/ 78%	WP_091080834.1/ GA0070616_RS11745	82%/ 73%	WP_091080797.1/ GA0070616_RS11675	84%/ 72%
Saccharothrix espanaensis	WP_015104204.1/ BN6_RS33070	81%/ 70%	WP_041314940.1 BN6_RS33065	72%/ 61%	WP_015103353.1/ BN6_RS28805	72%/ 58%

Supplementary Table 4 Oligonucleotide primers used in this study. The restriction sites are underlined, and the restriction enzymes are indicated in parentheses.

Primer	Sequence (5'- 3')
***rif15*-F**	AATCGCCATATGATGCAGATGACCGAAGAGAAC (*Nde*I)
***rif15*-R**	CCCAAGCTTGCCGACGAAGCGGGCGATCGA (*Hind*III)
***rif15a*-pSJ2 F**	CGCGGATCCCAGATGACCGAAGAGAACCT (*Bam*HI)
***rif15a*-pSJ2 R**	CCCAAGCTTTCATGCGGTGCTCCCTTCCT (*Hind*III)
***rif15b*-F**	GGAATTCCATATGACCGCCCAGGTGACCAGG (*Nde*I)
***rif15b*-R**	AGGAATTCTCAGCCGACGAAGCGGGCGATC (*Eco*RI)
***rif16*-F**	GGGAATTCCATATGGTGACGACCAAAGTGACC (*Nde*I)
***rif16*-R**	CCGCTCGAGTTAGGGAGCGTCCCAGGC (*Xho*I)

Supplementary Table 5 Data collection and refinement statistics for Rif16 structures

	**Rif16 native**	**R-L-bound Rif16**
**Data collection**		
**Space group**	p21	p21
**Cell dimensions**		
***a*, *b*, *c* (Å)**	35.12, 70.34, 81.02	35.08, 70.71, 80.975,
**a, b, g (°)**	90.0, 94.433, 90.0	90.0, 94.002, 90.0
**Resolution (Å)**	50.0-1.9 (1.94-1.90)	50.0-2.6 (2.64-2.60)
***R*sym or *R*merge**	0.092 (0.477)	0.110 (0.555)
I* / s*I	28.5 (7)	15.5 (3.13)
**Completeness (%)**	94.1 (95)	98.7 (99.3)
**Redundancy**	6.3 (6.4)	5.8 (5.7)
**Refinement**		
**Resolution (Å)**	1.9	2.6
**No. reflections**	33798	12806
**R_{work} / R_{free}**	0.182/0.229	0.211/0.274
**No. atoms**		
**Protein**	2804	2870
**Heme**	43	43
**Ligand**	no	54
**Water**	157	94
***B*-factors**		
**Protein**	22.99	37.95
**Heme**	11.42	23.15
**Ligand**	no	75.20
**Water**	28.23	33.90
**R.m.s. deviations**		
**Bond lengths (Å)**	0.019	0.018
**Bond angles (°)**	1.897	1.920

Highest-resolution shell is shown in parentheses.

Supplementary References

1 Omura, T. & Sato, R. The carbon monoxide-binding pigment of liver microsomes II. Solubilization, purification, and properties. *J. Biol. Chem.* **239**, 2379-2385 (1964).

2 Di Tommaso, P. *et al.* T-Coffee: a web server for the multiple sequence alignment of protein and RNA sequences using structural information and homology extension. *Nucleic Acids Res.* **39**, W13-17 (2011).

3 Robert, X. & Gouet, P. Deciphering key features in protein structures with the new ENDscript server. *Nucleic Acids Res.* **42**, W320-324 (2014).

4 Eddine, A. N. *et al.* X-ray structure of 4,4′-dihydroxybenzophenone mimicking sterol substrate in the active site of sterol 14α-demethylase (CYP51). *J. Biol. Chem.* **283**, 15152-15159 (2008).

5 Zhao, B. *et al.* Crystal structure of albaflavenone monooxygenase containing a moonlighting terpene synthase active site. *J. Biol. Chem.* **284**, 36711-36719 (2009).

6 Bell, S. G. *et al.* The crystal structures of 4-methoxybenzoate bound CYP199A2 and CYP199A4: structural changes on substrate binding and the identification of an anion binding site. *Dalton Trans.* **41**, 8703-8714 (2012).

7 Meharenna, Y. T. *et al.* Crystal structure of P450cin in a complex with its substrate, 1,8-cineole, a close structural homologue to d-camphor, the substrate for P450cam. *Biochemistry* **43**, 9487-9494 (2004).

8 Raag, R. & Poulos, T. L. Crystal structures of cytochrome P-450$_{CAM}$ complexed with camphane, thiocamphor, and adamantane: factors controlling P-450 substrate hydroxylation. *Biochemistry* **30**, 2674-2684 (1991).
